# Effects of Sinapic Acid Combined with Cisplatin on the Apoptosis and Autophagy of the Hepatoma Cells HepG2 and SMMC-7721

**DOI:** 10.1155/2021/6095963

**Published:** 2021-10-12

**Authors:** Jian Zhao, Hewei Li, Wanying Li, Zixian Wang, Zhewen Dong, Huan Lan, Chengqiang Wang, Jia-Le Song

**Affiliations:** ^1^Department of Pharmacology, School of Pharmaceutical Sciences, Guilin Medical University, Guilin 541004, China; ^2^Department of Infectious Diseases, School of Clinical Medicine, Guilin Medical University, Guilin 541004, China; ^3^Department of Nutrition and Food Hygiene, School of Public Health, Guilin Medical University, Guilin 541004, China; ^4^Department of Occupational and Environmental Health, School of Public Health, Guilin Medical University, Guilin 541004, China; ^5^Guangxi Health Commission Key Laboratory of Entire Lifecycle Health and Care, Guilin 541199, China; ^6^Department of Clinical Nutrition, The Second Affiliated Hospital of Guilin Medical University, Guilin 541004, China

## Abstract

Sinapic acid (Sa) is a small-molecule phenolic acid compound predominant in fruits, vegetables, and grains. This study investigated the antitumor effects of cisplatin (DDP) combined with Sa (Sa/DDP) on the hepatic cancer cells (HCC), HepG2 and SMMC-7721. The HepG2 and SMMC-7721 cells were treated with Sa or Sa/DDP, and the cell proliferation and cell cycle were detected using the MTT assay. The cell migration was detected using the transwell and scratch assays, while apoptosis and autophagy were detected using Hoechst, MDC, and Annexin V-FITC/PI staining. The protein expression was quantitated using the western blot. Sa/DDP was found to not only inhibit cancer cell proliferation and migration but also induce cell apoptosis. Simultaneously, the Sa/DDP combination was found to activate autophagy, and the HCQ autophagy inhibitor enhanced the apoptosis in the Sa/DDP-induced liver cancer cells. The combined use of Sa and DDP makes it an attractive adjuvant therapy strategy for tumors, establishing the prospect of phenolic acid compounds for the adjuvant treatment of hepatocellular carcinoma.

## 1. Introduction

Hepatocellular carcinoma (HCC) is one of the several malignancies posing a severe threat to human health and accounting for more than 85% of all primary liver cancer [1]. Cisplatin (DDP) is the first-line drug for treating various solid tumors. After entering the cell, DDP mainly targets the DNA to abrogate the mitotic progression. It can also disrupt the typical double-helical structure of DNA, inactivating and denaturing it locally, and finally induce apoptosis [2]. However, due to various factors, patients with liver cancers are resistant to the antitumor drugs, which compromises the therapeutic potential [3–5]. Therefore, studies on the combination therapy of cisplatin may serve as the treatment strategy to resolve clinical adverse reactions and adverse prognoses.

**Figure 1:**
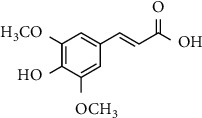
The chemical structure of sinapic acid.

Fruits and vegetables are enriched with a large number of phenolic acids [6]; therefore, increasing the intake of these foods tends to reduce the risk of cancer. Advanced knowledge on such fruits and vegetables can, therefore, improve the prognosis of colon cancer, esophageal cancer, lung cancer, oral cancer, and gastric cancer, respectively [7]. Small-molecule phenolic acids are widely used for preventing and treating various tumors due to their bioactive solid components [8, 9]. Combining the phenolic acid compounds with the standard chemotherapeutic drugs shows effective antitumor activity. Caffeic acid combined with DDP can effectively inhibit the growth of the HeLa cells in cervical cancer [10]. The combination of caffeic acid with DDP may also hinder the spread of the A2780 ovarian cancer cells [11]. The combination of rosmarinic acid with DDP has a sensitization effect on the DDP-resistant A549 lung cancer cells [12]. Based on these reports, a combination of phenolic acid with the standard chemotherapy drugs is hypothesized to have an antitumor role. Sinapic acid (Sa) is a natural hydroxycinnamic acid derivative (Figure 1), with multiple pharmacological activities such as anti-inflammatory, antibacterial, anticancer, and scavenging oxygen free radicals. However, the antitumor effects of Sa combined with DDP (Sa/DDP) on the HepG2 and SMMC-7721 cells and the impact of the Sa/DDP combination on autophagy and apoptosis are still unclear. This study aims to reveal the role of Sa/DDP in treating hepatic cancer and further explores its influence on autophagy and apoptosis. It may serve as an experimental basis for improving the anticancer effect of the chemotherapeutic drugs in combination with Sa.

## 2. Materials and Methods

### 2.1. Chemical Reagents

Sa (S106903, purity ≥98%) was obtained from Aladdin Biochemical Technology Co., Ltd. (Shanghai, China). DDP (SC5170, purity ≥98%) and the monodansylcadaverine (MDC) staining kit (G1070) were purchased from Solarbio Science and Technology Co., Ltd. (Beijing, China). Hydroxychloroquine sulfate (HCQ; H0915, purity ≥98%) and Z-VAD-FMK (219007, purity ≥98%) were purchased from the Sigma-Aldrich Chemical Company (Sigma, St. Louis, MO, USA). The cell cycle and apoptosis analysis kit (C1052), Hoechst staining kit (C0003), Annexin V-fluorescein-isothiocyanate (FITC)/propidium iodide (PI) apoptosis detection kit (C1062), BeyoECL plus (P0018), BCA protein assay kit (P0012), HRP-labeled goat anti-rabbit IgG (H + L) (A0208) and rabbit anti-B-cell lymphoma-2(Bcl-2; AF0060), apoptosis regulator Bcl-2-like protein 4 (Bax; AF0057) polyclonal antibody and rabbit anti-poly ADP-ribose polymerase (PARP; AF1657), cleaved-PARP (c-PARP; AF1567), cysteine-requiring aspartate protease-3 (caspase-3; AF1675), active-caspase-3 (a-caspase-3; AF1150), and autophagy protein 5 (Atg5; AF2269) monoclonal antibodies were procured from Beyotime Institute of Biotechnology (Shanghai, China). The rabbit anti-*β*-actin (GB11001), sequestosome-1 (p62; GB11531) polyclonal, microtubule-associated proteins 1A/1B light chain 3 (LC3I/II and GB11124), and Beclin-1 (GB3228-1) polyclonal antibody were procured from Servicebio Co., Ltd. (Wuhan, China).

### 2.2. Cell Culture

The human hepatic line, HepG2 (KCB200507YJ), was derived from the Cell Bank of the Chinese Academy of Sciences (Kunming, China). The human HCC cell line SMMC-7721 (TCHu52) and normal liver cell line HL-7702 (GNHu6) were procured from the Chinese Academy of Science Type Culture Collection (Shanghai, China). The cells were cultured in Dulbecco's modified Eagle's medium (DMEM; Gibco, Gran Island, USA) containing 10% (v/v) fetal bovine serum (FBS; Gibco), 100 U/mL penicillin, and 100 *µ*g/mL streptomycin sulfate. All the cells were incubated in a humidified incubator (3111, Thermo Fisher Scientific Inc., USA) containing 5% CO_2_ at 37°C.

### 2.3. Cell Viability Assay

The cells HepG2, SMMC-7721, and HL-7702 were seeded in 96-well plates containing 6,000 cells per well, supplemented with various concentrations of Sa (0–2,000 *μ*M) and DDP (5 *μ*M) [13]. After the cells were incubated at 37°C for 24, 48, and 72 h, they were cultured with 20 *µ*L MTT (5 mg/mL) for 4 h. The formazan crystals were dissolved by adding 150 *µ*L of dimethyl sulfoxide, followed by recording the absorbance at 490 nm using a microplate reader (Varioskan LUX, Thermo Fisher Scientific Inc., USA) and calculating the percentage of cell viability.

### 2.4. Colony Formation Assay

The HepG2 and SMMC-7721 cells were seeded in a 12-well plate (200–300 cells/well). Based on the experimental grouping, the cells were coincubated with 0–600 *µ*M Sa and 5 *µ*M DDP DMEM (1 mL) for 72 h. Then, the recovery medium was added to the complete medium (1 mL) and cultured for 10 d. Subsequently, these cells were fixed in 4% paraformaldehyde solution (1 mL) at 37°C for 2 h, stained with 0.5% crystal violet staining solution (0.5 mL) for 15 min, and photographed, and the numbers of colonies formed were tabulated. The findings were expressed as a percentage of the number of colonies in each experimental group (assuming that the control group was 100%). The ImageJ software (Rawak Software Inc., Stuttgart, Germany) was used for analyzing the data.

### 2.5. Cellular Morphology Studies and Cell Cycle Analysis

The HepG2 and SMMC-7721 cells were seeded in a 6-well plate (5 × 10^5^ cells/well). The cells were photographed using the inverted microscope (Eclipse Ti-S, Nikon Co., Ltd., Japan) to observe the morphological changes. The cells were collected, treated with 70% precooled ethanol (1 mL) in an ice bath, blew and mixed well, and then, fixed overnight at 4°C. The cells were stained with 25 *µ*L PI staining solution and 10 *µ*L RNase staining solution, according to the manufacturer's protocol, and the cell cycle was analyzed by flow cytometry (Attune NxT, Thermo Fisher Scientific Inc., USA).

### 2.6. Transwell Assay

The HCC cells (3 × 10^4^ cell/well) were incubated in 200 *μ*L DMEM (containing 1% FBS) with 0–600 *µ*M Sa and 5 *µ*M DDP as the upper chamber. The lower section contained 700 *μ*L of DMEM (including 10% FBS) along with the drug to be loaded. Briefly, 3 × 10^4^ cells were added to the upper chamber while the lower chamber was filled with 10% FBS after incubating the cells for 24 h. The cells were cultured at 37°C for 24 h, fixed with a 4% paraformaldehyde solution, and stained with 0.5 mL of crystal violet. Subsequently, the cells in the upper chambers were removed by gently dabbing them with cotton swabs. These cells were photographed, and the invaded cells were observed using an inverted microscope (DM4 B, Leica Inc., Germany). Finally, the stained cells were counted in five randomly chosen fields under the microscope for further analysis.

### 2.7. Wound Healing Assay

The HepG2 and SMMC-7721 cells were seeded in a 6-well plate (5 × 10^5^ cells/well) and allowed to grow until confluent. The layer of fusion cells was delicately scratched with the tip of a 200 *μ*L pipette. The cells were washed three times with the phosphate-buffered saline (PBS) and cultured in DMEM containing 1% FBS and 0–600 *µ*M Sa and 5 *µ*M DDP for 24 h. The wound images were captured at 0 and 24 h using an inverted microscope (DM4 B, Leica Inc., Germany), respectively. The extent of the wound closing was estimated as the percentage of wound healing.

### 2.8. Hoechst Staining Assay

The HepG2 and SMMC-7721 cells were seeded into 24-well plates (2 × 10^5^ cell/well) and incubated until the cells adhered. The cells were coincubated with 0–600 *µ*M Sa and 5 *µ*M DDP for 72 h. Then, according to the manufacturer's protocol, the cell was washed thrice with PBS. These cells were then fixed in 4% paraformaldehyde solution for 2 h, at 20°C, followed by rinsing with PBS three times and adding Hoechst staining solution to stain for 10 min with rinsing thrice. These cells were then photographed and observed under a Leica fluorescence microscope (excitation wavelength at 350 nm and emission wavelength at 460 nm).

### 2.9. Annexin V-FITC/PI Staining Assay

The HepG2 and SMMC-7721 cells were seeded into a 6-well plate (1 × 10^5^ cell/well), and after 72 h, the cells in each group were treated with trypsin and collected. The cells were rinsed twice in PBS, and a concentration of 5 × 10^5^ cells/mL was treated with a binding buffer. According to the manufacturer's protocol, the cells were stained with 5 *µ*L PI staining solution and 10 *µ*L Annexin V-FITC staining solution with incubation for 30 min in the dark. These cells were then analyzed by flow cytometry (Attune NxT, Thermo Fisher Scientific Inc., USA) using excitation/emission wavelengths of 488/525 nm and 488/675 nm for Annexin V-FITC and PI, respectively.

### 2.10. MDC Staining Assay

The HepG2 and SMMC-7721 cells were seeded into a 96-well plate at the rates of 5,000 cells per well and treated with different concentrations of Sa for 72 h followed by rinsing with the washing buffer. According to the manufacturer's instructions, the cells were stained with 10 *µ*L MDC staining solution in the dark and incubated at 37°C for 30 min. These cells were then observed and photographed under a Nikon fluorescence microscope (excitation filter wavelength 355 nm and blocking filter wavelength 512 nm).

### 2.11. Total Protein Collection and Western Blotting Assay

The HepG2 and SMMC-7721 cells treated with Sa/DDP were collected and using radioimmunoprecipitation assay buffer to lyse the cells at 4°C for 30 min to extract the total protein. The protein concentration of the samples was measured using the BCA protein assay kit, after centrifugation at 12,000 g at 4°C for 5 min, and denatured by boiling at 100°C. An equal amount of protein (30 *μ*g) was resolved using the sodium dodecyl sulfate-polyacrylamide gel (10–12%) electrophoresis for 1.5 h. The proteins were then transferred onto a membrane of nitrocellulose and blocked using 5% skimmed milk for 2 h at 20°C, followed by rinsing thrice using PBS containing 0.1% Tween 20 (PBS-T), for 5 min each. Furthermore, the cells were incubated in diluted anti-Bax, Bcl-2 PARP, c-PARP, a-caspase-3, caspase-3, p62, Beclin-1, Atg5, LC3I/II, and *β*-actin according to 1 : 1,000, incubated overnight at 4°C, and subsequently washed repeatedly the next day. The membranes were incubated with a secondary antibody (1 : 3000) for 1 h at ambient temperature and washed repeatedly. After washing the membrane, it was developed by the FluorChem M image acquisition system (Protein Simple Inc., USA). The quantification was performed (where appropriate) using the ImageJ software (Rawak Software Inc., Stuttgart, Germany).

### 2.12. Statistical Analysis

In this study, data from at least three independent experiments were obtained and represented as mean ± standard deviation. All the statistical analyses were performed using the SPSS version 25.0 (SPSS Inc., Chicago, IL, USA). The essential differences between the different groups were analyzed using the unidirectional variance analysis (ANOVA) and Duncan's multiple range test. *P* < 0.05 was considered to be significant. All the analytical figures were plotted using the GraphPad Prism 8.0 software (GraphPad Software Inc., San Diego, CA, USA).

## 3. Results

### 3.1. Sa/DDP Inhibits the HCC Cell Proliferation

**Figure 2:**
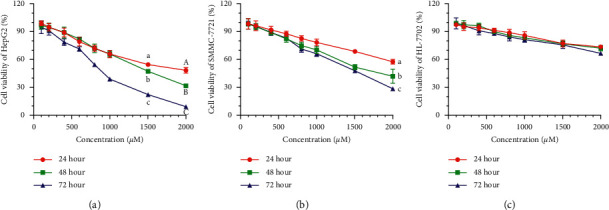
The cell viability of (a) HepG2, (b) SMMC-7721, and (c) HL-7702 cells coincubated with 0–2,000 *µ*M of Sa for 24 h, 48 h, and 72 h. All results were exhibited as the means ± SD of five different samples. ^a–c, A–C^Bars with different superscript letters differ significantly (*P* < 0.05) by Duncan's multiple range test.

**Figure 3:**
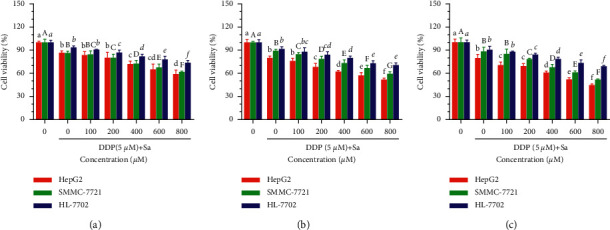
The cell viability of HepG2, SMMC-7721, and HL-7702 cells coincubated with 0–800 *µ*M of Sa and 5 *µ*M DDP for (a) 24 h, (b) 48 h, and (c) 72 h. All results were exhibited as the means ± SD of five different samples. ^a–f, A–G,^^*a–f*^Bars with different superscript letters differ significantly (*P* < 0.05) by Duncan's multiple range test.

To determine the antitumor activity of Sa, the HepG2, SMMC-7721, and HL-7702 cells were selected as in vitro models. The MTT results showed that Sa (0–2000 *μ*M) inhibited the growth of the HCC cells as a function of concentration and time (Figure 2). After the treatment with Sa (600 *µ*M) for 72 h, the viability of the HepG2 cells decreased to 71.26 ± 4.08% and the viability of HepG2 cells decreased to 51.77 ± 3.68% after combining with DDP (Figure 3). However, the inhibitory effect on the normal liver HL-7702 cells was not pronounced. It showed that Sa enhances the sensitivity of DDP to HCC cells and produces an antiproliferation impact. Based on the half maximal inhibitory concentration (IC50) values for 72 h, the Sa concentrations of 200 *μ*M, 400 *μ*M, and 600 *μ*M were selected for subsequent experiments (Tables 1 and 2).

**Figure 4:**
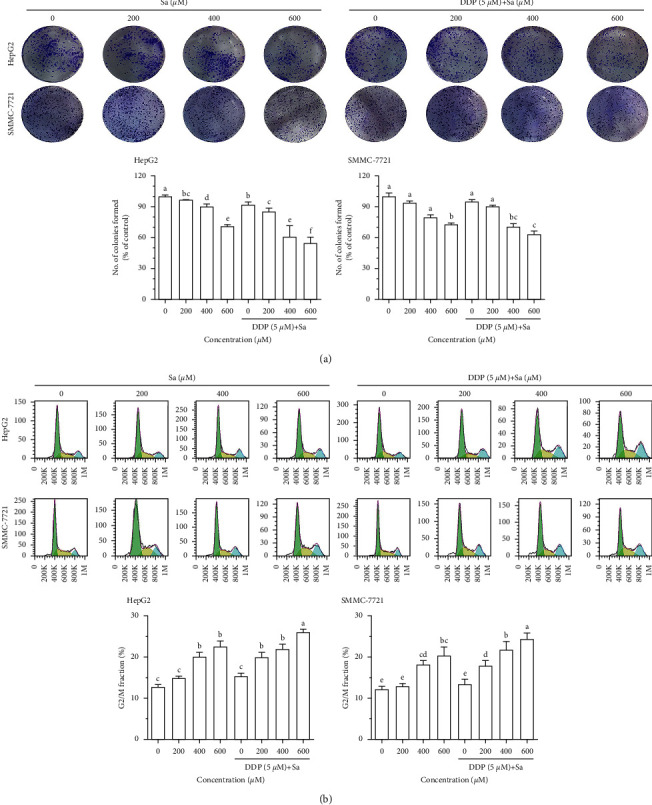
Sa/DDP inhibits the proliferation of the HepG2 and SMMC-7721 cells. (a) The proliferation ability of the HepG2 and SMMC-7721 cells coincubated with Sa (0, 200, 400, and 600 *µ*M) and 5 *µ*M DDP for 72 h was evaluated through the colony formation assay. (b) The cell cycle changes of the HepG2 and SMMC-7721 cells coincubated with Sa (0, 200, 400, and 600 *µ*M) and 5 *µ*M DDP for 72 h were evaluated by flow cytometry. All results were exhibited as the means ± SD of three different samples. ^a–f^Bars with different superscript letters differ significantly (*P* < 0.05) by Duncan's multiple range test.

To assess the effect of Sa/DDP on the HCC cell proliferation, a 10-day colony formation assay was performed (Figure 4(a)). Sa was found to decrease the proliferation of the HCC cells, and the cell proliferation activity was further reduced after being combined with DDP. Moreover, the most significant inhibition of proliferation was obtained at the highest concentration of Sa (600 *μ*M)/DDP (5 *μ*M). Compared to the control group, the proliferation activity in the HepG2 and SMMC-7721 cells declined to 54.96 ± 9.55% and 63.23 ± 2.9%, respectively. After Sa (600 *μ*M) treatment, the proliferation activities of HepG2 and SMMC-7721 cells were 71.46 ± 2.23% and 72.90 ± 1.29%, respectively, which were statistically different compared with combined DDP. These results indicated that Sa treatment induces a proliferation inhibitory effect on the HCC cells and combined with DDP can enhance the antitumor effect of Sa.

Then, the effect of Sa/DDP was examined on the cell cycle of the HCC cells (Figure 4(b)). The results demonstrated that the cell cycle was blocked in the G2/M phase after Sa treatment and was concentration dependent (*P* < 0.05). The percentage of the HepG2 cells in the G2/M phase after Sa (600 *μ*M) treatment increased from 12.79 ± 0.89% in the control group to 22.60 ± 2.26% after treatment. In addition, compared to the DDP (5 *μ*M) group (15.40 ± 1.23%), there was an increase in the percentage of cells in the G2/M phase in the case of the Sa (600 *μ*M)/DDP (5 *μ*M) group (26.00 ± 1.30%). Also, the Sa (600 *μ*M)/DDP (5 *μ*M) group is statistically significant compared to the Sa (600 *μ*M) group. It could be concluded that the Sa with DDP combination can increase the G2/M phase block ratio of HCC cells and enhance the anti-HCC effect of DDP.

### 3.2. Sa/DDP Inhibits the HCC Cell Migration

**Figure 5:**
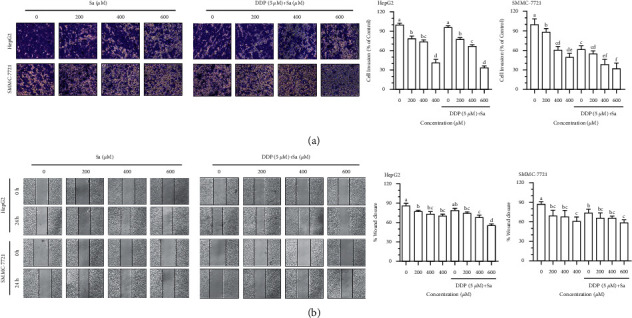
Sa/DDP inhibits the migration of the HepG2 and SMMC-7721 cells. (a) The migration ability of the HepG2 and SMMC-7721 cells coincubated with Sa (0, 200, 400, and 600 *µ*M) and 5 *µ*M DDP for 24 h through the transwell assay (40x magnification) was evaluated. (b) The migration ability of the HepG2 and SMMC-7721 cells coincubated with Sa (0, 200, 400, and 600 *µ*M) and 5 *µ*M DDP for 24 h was evaluated by the scratch assay (10x magnification). All results were exhibited as the means ± SD of three different samples. ^a–f^Bars with different superscript letters differ significantly (*P* < 0.05) by Duncan's multiple range test.

Tumor metastasis is a significant problem in the current tumor treatment, and liver cancer is characterized by early metastasis, so cell migration is an essential step in the progression of malignant tumors. To explore the effect of Sa/DDP on the migration of the HepG2 and SMMC-7721 cells, the cell migration rate was tested through the transwell and wound healing experiments (Figure 5(a)). The results showed that Sa/DDP can inhibit the migration of the HCC cells as evident from the decrease of the migration rate of the HepG2 and SMMC-7721 cells by 41.52 ± 8.80% and 49.80 ± 5.78%, respectively, after Sa (600 *μ*M) treatment. After using DDP, the mobility of the HepG2 and SMMC-7721 cells was further reduced by 33.94 ± 4.45% and 32.34 ± 8.12%. Hence, it could be concluded that Sa/DDP treatment of the HCC cells can effectively inhibit cell migration. We had observed a similar phenomenon in the subsequent wound healing experiments (Figure 5(b)).

### 3.3. Sa/DDP Induces Apoptosis of the HCC Cells

**Figure 6:**
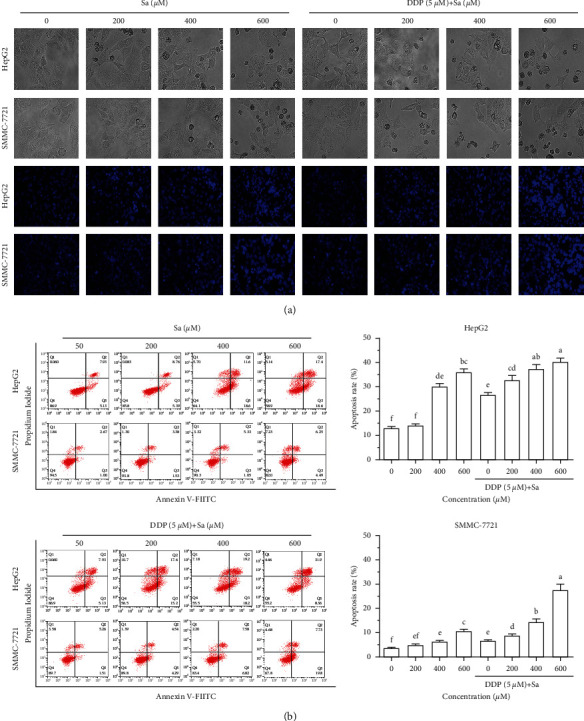
Sa/DDP induces the apoptosis of the HepG2 and SMMC-7721 cells. (a) The HepG2 and SMMC-7721 cells coincubated with Sa (0, 200, 400, and 600 *µ*M) and 5 *µ*M DDP for 72 h were observed for the morphological changes and the Hoechst (blue) staining by using a fluorescence microscope (40x magnification). (b) The percentage of apoptosis of HepG2 and SMMC-7721 cells coincubated with Sa (0, 200, 400, and 600 *µ*M) and 5 *µ*M DDP for 72 h was evaluated by Annexin V-FITC/PI staining and flow cytometric analysis. All results were exhibited as the means ± SD of three different samples. ^a–f^Bars with different superscript letters differ significantly (*P* < 0.05) by Duncan's multiple range test.

To determine the mode of cellular death induced by Sa, the effect of various concentrations of Sa/DDP was observed on the morphology of the HCC cells. The HCC cells in the control group were normal in morphology, spindle shaped with well-defined extensions (Figure 6(a)). After Sa treatment, the cells had undergone shrinkage, were rounded, losing their normal cell morphology, and underwent apoptosis. To verify the appearance of the apoptotic cells, we found that the HepG2 and SMMC-7721 cells that underwent apoptosis showed chromatin aggregation, concentration, and nucleus fragmentation and an increase in the number of apoptotic cells with the increasing drug concentration. In contrast, only few cells in the control group underwent apoptosis. The abovementioned results indicate that the cells treated with Sa/DDP led to morphological changes in apoptosis. Concomitantly, the Annexin V-FITC/PI results showed that Sa/DDP treatment could significantly increase the apoptotic rate of the HCC cells (Figure 6(b)). The apoptotic rates of the HepG2 and SMMC-7721 cells in the control group were 13.06 ± 1.04% and 3.67 ± 0.34%, respectively. Furthermore, after Sa (600 *μ*M) treatment, the apoptosis rate increased to 36.00 ± 2.52%, 10.74 ± 0.75%, but when combined with DDP, the apoptotic rate was increased by 40.26 ± 2.82% and 27.53 ± 2.48%, respectively. This suggested that Sa/DDP can induce the apoptosis of the HCC cell. Compared to Sa or DDP alone, Sa/DDP was found to increase the apoptosis rate of the HCC cells and enhances its antitumor effect.

**Figure 7:**
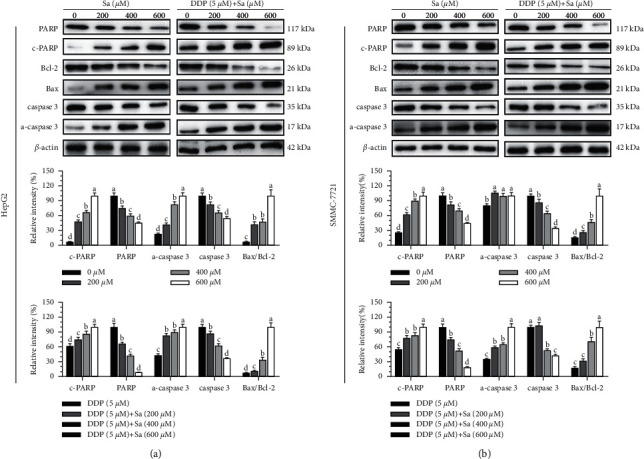
The Sa/DDP treatment induces apoptosis of the HepG2 and SMMC-7721 cells. (a, b) Western blot was used to analyze the expression in the HepG2 and SMMC-7721 cells coincubated with Sa (0, 200, 400, and 600 *µ*M) and 5 *µ*M DDP for 72 h of apoptosis-related proteins PARP, Bcl-2, Bax, and caspase-3, with *β*-actin as the internal reference. All results were exhibited as the means ± SD of three different samples. ^a–d^Bars with different superscript letters differ significantly (*P* < 0.05) by Duncan's multiple range test.

To further explore the mechanism of Sa/DDP-induced apoptosis, the expression of the proteins associated with apoptosis was measured through western blot. The cleaved PARP and caspase-3 in the HCC cells were found to be activated by the interventional treatment with Sa or DDP (Figure 7). In the HepG2 cells, the Sa (600 *μ*M) treatment compared to the control group, the cleaved PARP and caspase-3 protein increased 4.3 times and 13.6 times, respectively, and the lysis phenomenon was more evident after combination with DDP. The increased proportion of Bax/Bcl-2 plays a critical role in activating apoptosis. We observed an increase in the expression of the proapoptotic Bax protein and a decrease in the expression of the antiapoptotic Bcl-2 protein. The Bax/Bcl-2 ratio was proportional to the dose of the drug (*P* < 0.05). These data were consistent with the Hoechst staining and Annexin V-FITC/PI results, indicating the activation of an apoptotic signaling pathway. Moreover, it was suggested that Sa can induce the apoptosis of the HCC cells through the Bax/Bcl-2 pathway. Meanwhile, in the HepG2 cells, compared to DDP alone, the expression of c-PARP and a-caspase-3 increased by 38.22 ± 1.39% and 57.30 ± 2.81%, respectively. A similar situation was found in the SMMC-7721 cells, where the expression levels of c-PARP and a-caspase-3 were found to increase by 44.68 ± 3.64% and 64.27 ± 5.66%, respectively.

### 3.4. Sa/DDP Induces Autophagy in the HCC Cells

**Figure 8:**
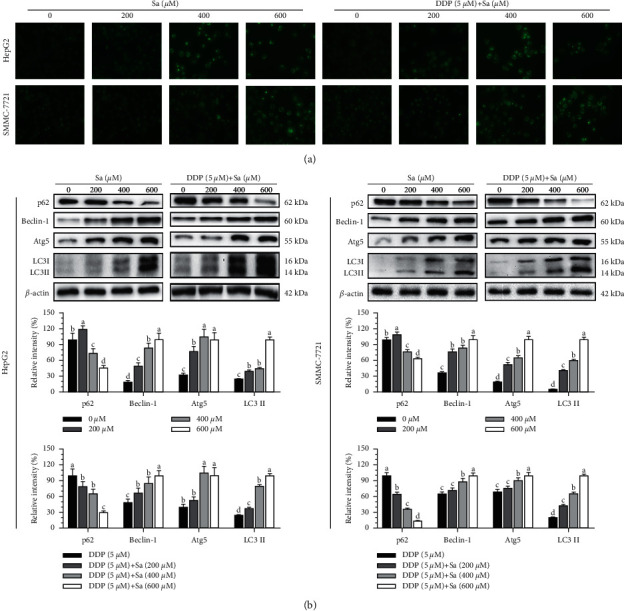
The Sa/DDP treatment induces autophagy of the HepG2 and SMMC-7721 cells. (a) The HepG2 and SMMC-7721 cells coincubated with Sa (0, 200, 400, and 600 *µ*M) and 5 *µ*M DDP for 72 h MDC (green) staining were observed by using a fluorescence microscope (40x magnification). (b) Western blot was used to analyze the expression in the HepG2 and SMMC-7721 cells coincubated with Sa (0, 200, 400, and 600 *µ*M) and 5 *µ*M DDP for 72 h of the autophagy-related proteins p62, Beclin-1, Atg5, and LC3 II, with *β*-actin as the internal reference. All results were exhibited as the means ± SD of three different samples. ^a–d^Bars with different superscript letters differ significantly (*P* < 0.05) by Duncan's multiple range test.

To explore whether Sa can induce the death of the HepG2 and SMMC-7721 cells by activating autophagy, the MDC staining was used to detect autophagy. Sa treatment was shown to increase the number of green granular structures in the cytoplasm and nucleus area of the HepG2 and SMMC-7721 cells compared to the control group, suggesting that Sa inhibits the activity of the HCC cells with simultaneous activation of the intracellular autophagy reaction (Figure 8(a)). LC3 is a marker protein on the autophagosome membrane such that when intracellular autophagy is activated, LC3I is transformed into LC3II. The content of LC3II is directly proportional to the number of autophagic vacuoles. The western blot results showed that the expression of intracellular LC3II protein was significantly increased in a dose-dependent manner (*P* < 0.05), suggesting that Sa treatment can induce autophagy in the HCC cells (Figure 8(b)). The Sa treatment was concomitantly found to promote the expression of Beclin-1 and Atg5 in the cells and reduce the expression of p62. When the HepG2 cells were treated with Sa (600 *μ*M) for 72 h, the protein expression of Beclin-1 and Atg5 increased by 4.8-fold and 2.9-fold, while the expression of p62 protein decreased by 2.3-fold. At the same time, in the SMMC-7721 cells, the Beclin-1 and Atg5 expression increased by 2.5 times and 4.7 times and the p62 expression decreased by 1.7 times. The Sa/DDP treatment particularly showed a similar trend such that when the HepG2 cells were treated with Sa (600 *μ*M)/DDP (5 *μ*M), the Beclin-1 and Atg5 expression increased by 2 times and 2.4 times compared to that in DDP alone. In the SMMC-7721 cells, the Beclin-1 and Atg5 proteins were increased by 1.4 times and 1.3 times, and the expression of p62 decreased by 7.7 times in the SMMC-7721 cells. It could, therefore, be suggested that Sa/DDP treatment can induce autophagy in the HepG2 and SMMC-7721 cells, and its strength has a dose-effect relationship with the drug concentration.

### 3.5. The Autophagy Inhibitor Enhances the HCC Cell Apoptosis Induced by Sa/DDP

**Figure 9:**
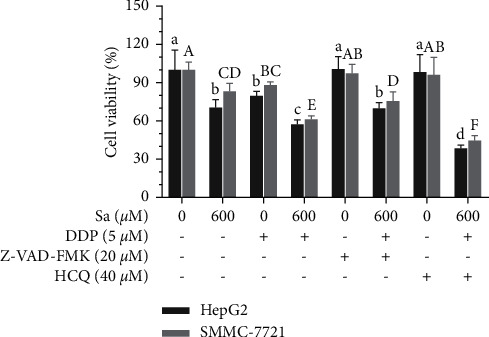
Inhibition of autophagy reduces the viability of the HepG2 and SMMC-7721 cells. The hepatic cancer cells were pretreated with the apoptosis inhibitor 20 *µ*M Z-VAD-FMK or autophagy inhibitor 40 *µ*M HCQ for 1 h. The cell viability of the HepG2 and SMMC-7721 cells coincubated with Sa (0 and 600 *µ*M) and 5 *µ*M DDP for 72 h was evaluated by the MTT assay. All results were exhibited as the means ± SD of five different samples. ^a–d, A–F^Bars with different superscript letters differ significantly (*P* < 0.05) by Duncan's multiple range test.

Apoptosis and autophagy are closely related irrespective of being complimentary or antagonistic, and both of them regulate cell survival. To describe the relationship between autophagy and apoptosis, the effects of the apoptotic inhibitor Z-VAD-FMK and autophagy inhibitor HCQ were analyzed in the Sa/DDP-induced cell death. The cell viability of the HepG2 cells pretreated with Z-VAD-FMK (20 *μ*M) and HCQ (40 *μ*M) for 1 h and without drug treatment was determined to be 100.66 ± 12.14%, 98.29 ± 18.68%, and 100.00 ± 19.04%, respectively (Figure 9). Therefore, it is believed that the pretreatment of Z-VAD-FMK (20 *μ*M) and HCQ (40 *μ*M) does not significantly affect the viability of the HepG2 and SMMC-7721 cells. Compared to Sa/DDP (57.40 ± 4.54%), Z-VAD-FMK pretreatment with Sa/DDP was found to increase the cell viability by 12.65 ± 7.97%. In comparison, the HCQ pretreatment was found to reduce the cell viability by 19.07 ± 4.70%, and SMMC-7721 cells also show similar trends suggesting that HCQ and Sa/DDP treatment of the liver cancer cells can inhibit the tumor cell viability more effectively.

**Figure 10:**
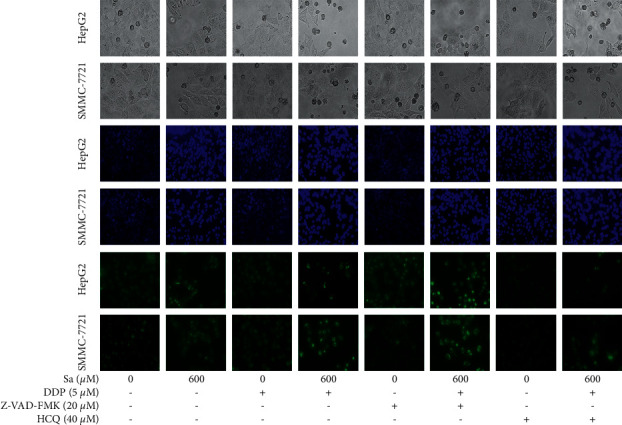
Inhibition of autophagy enhances the Sa/DDP-induced apoptosis of the HepG2 and SMMC-7721 cells. The hepatic cancer cells were pretreated with the apoptosis inhibitor 20 *µ*M Z-VAD-FMK or the autophagy inhibitor 40 *µ*M HCQ for 1 h. The HepG2 and SMMC-7721 cells coincubated with Sa (0 and 600 *µ*M) and 5 *µ*M DDP for 72 h were observed for morphological changes and Hoechst (blue) staining MDC (green) staining by using a fluorescence microscope (40x magnification).

To study the effects of Z-VAD-FMK and HCQ pretreatment on the autophagy and apoptosis of the HepG2 and SMMC-7721 cells induced by Sa/DDP, the cells were visualized under the light microscope. The control group without drug treatment and Z-VAD-FMK and HCQ pretreatment showed normal cell morphology and good ductility (Figure 10). The Hoechst staining showed weak fluorescence intensities after Z-VAD-FMK treatment and the HCQ treatment. It is suggested that Z-VAD-FMK (20 *μ*M) and HCQ (40 *μ*M) can inhibit apoptosis and autophagy, respectively. In the cells treated with Sa/DDP, the abnormal cell morphology could be seen after HCQ treatment, showing shrinkage and rounding, and a large number of apoptotic cells appeared in the visual field, suggesting that apoptosis plays a crucial role in the death of the HCC cells induced by Sa/DDP.

**Figure 11:**
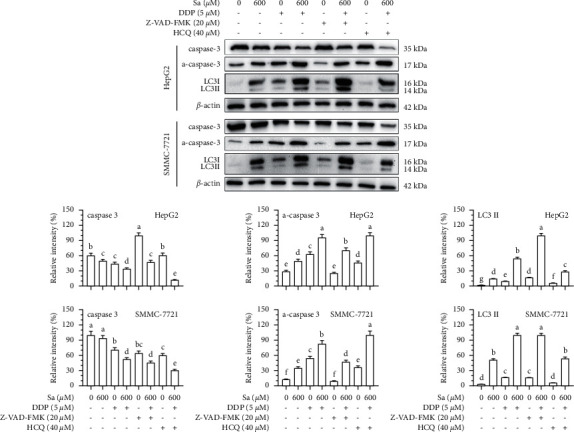
Inhibition of autophagy enhances the Sa/DDP-induced apoptosis of the HepG2 and SMMC-7721 cells. The hepatic cancer cells were pretreated with the apoptosis inhibitor 20 *µ*M Z-VAD-FMK or autophagy inhibitor 40 *µ*M HCQ for 1 h. Western blot was used to analyze the expression in the HepG2 and SMMC-7721 cells coincubated with Sa (0 and 600 *µ*M) and 5 *µ*M DDP for 72 h of apoptosis-related proteins caspase-3 and autophagy-related proteins LC3II, with *β*-actin as the internal reference. All results were exhibited as the means ± SD of three different samples. ^a–g^Bars with different superscript letters differ significantly (*P* < 0.05) by Duncan's multiple range test.

To further explore the effect of Sa/DDP on apoptosis and autophagy of the HepG2 and SMMC-7721 cells at a molecular level, the caspase-3, and LC3II proteins were detected, respectively, and the Sa/DDP treatment was found to increase the caspase-3 protein cleavage (Figure 11). At the same time, the presence of Z-VAD-FMK improved this situation. In the HepG2 cells, compared to the group without Z-VAD-FMK, the caspase protein lysis decreased by 25.22 ± 1.22%, respectively, while the expression of LC3II protein increased by 45.30 ± 2.05%. These results showed that the inhibition of apoptosis can enhance autophagy induced by Sa/DDP. Pretreatment with HCQ increased the cleavage of the caspase-3 protein to a greater extent than when the cells were exposed to Sa/DDP. Compared to the Sa/DDP treatment, the expression of the a-caspase-3 protein in the HepG2 and SMMC-7721 cells increased 0.34 times and 0.73 times, respectively. In addition, HCQ decreased the expression of LC3II protein, suggesting that the inhibition of autophagy can enhance the Sa-induced apoptosis.

## 4. Discussion

Phenolic acid compounds can enhance the influence of chemotherapy drugs and protect the normal cells upon the treatment of HCC [14–16]. Sa simultaneously has the therapeutic potential in prostate cancer, lung cancer, breast cancer, and colon cancer [17–19]. However, the effect of DDP on the HCC has not been evaluated in detail. Therefore, we studied whether Sa/DDP can induce a better antitumor effect on the HCC cells. Sa was found to enhance the inhibitory effect of DDP on the HepG2 and SMMC-7721 cells, while the combined treatment was found to have a less toxic effect on the normal liver HL-7702 cells. Sa can inhibit the colon cancer SW480 cells and human breast cancer MDA-MB-468 cells [19], while caffeic acid can significantly enhance the antitumor activity of adriamycin on the breast cancer MCF-7 cells [20]. Ming et al. found that corilagin can inhibit the proliferation of the liver cancer SMMC-7721 and Bel-7402 cells and block the cell cycle in the G2/M phase [21]. Gambogic acid inhibited the proliferation of the tumor cells by blocking the Hep3B cells in the G0/G1phase [22]. The Sa/DDP treatment was found to check the cell cycle in the G2/M phase and prevent the HCC cells from undergoing mitosis, thus inhibiting their proliferation. This may be related to the DNA damage of the HCC cells caused by drugs, leading to the initiation of the cell cycle examination sites [23]. Tumor metastasis has always been a difficult point in treating tumors clinically. Clinical data show that 80% of tumor patients die due to tumor metastasis [24]. Some studies reported Sa to inhibit the migration of the prostate cancer PC-3 and LNCaP cells [17]. Caffeic acid can inhibit the migration of breast cancer MCF-7 cells [25, 26]. Similarly, in our study, Sa/DDP treatment was found to inhibit the HCC cell migration, suggesting that Sa/DDP treatment demonstrates antitumor activity.

Inducing the apoptosis of the tumor cells to a reasonable extent is an effective means in tumor treatment. The caspase protein family is crucial for regulating apoptosis, and caspase-3 activation is an important indicator of cell apoptosis [27]. PARP is considered a receptor of DNA damage, which can recognize and repair the broken DNA fragments, and its increased lysis indicates apoptosis in cells [28]. Some studies suggest the small phenolic acids induce apoptosis of the tumor cells. Sa [17] and gallic acid [29] can increase the Bax and caspase-3 in the prostate cancer PC-3 and LNCaP cells and induce their apoptosis. Gambogic acid can induce the apoptosis of various pancreatic cancer cells by upregulating the levels of caspase-3 and PARP protein cleavage [30]. We found Sa/DDP to interfere with the HepG2 and SMMC-7721 cells. With the increase in the drug concentration, the apoptosis phenomenon became more pronounced, with a significant increase in the expression of c-PARP and a-caspase-3 protein. The expression of the PARP and caspase-3 protein was decreased after the intervention. Although many proteins can regulate apoptosis through cascade reactions, the Bax/Bcl-2 ratio plays a vital role in regulating apoptosis [31]. Caffeic acid phenethyl ester can control the balance between Bax/Bcl-2 and induce the apoptosis of the ovarian cancer OV7 cells to play an antitumor role [32]. Gambogic acid [30, 33] and gallic acid [29] can upregulate the Bax protein expression and downregulate the Bcl-2 protein expression, inducing tumor cell apoptosis. This study found Sa/DDP to activate the apoptosis signal pathway and kill the HCC cells by upregulating the Bax protein expression and downregulating the Bcl-2 protein expression.

Autophagy is a process of clearing the aging organelles and recycling the intracellular substances, playing an essential role in maintaining the homeostasis of the intracellular environment [34]. The activation mechanism of autophagy in the tumor cells involves the upregulation of Atg5, Beclin-1, and LC3II and downregulation of p62 protein expression [35]. Salvianolic acid B can induce the autophagic death of the liver cancer SK-Hep1 and Bel-7402 cells [36]. In the usnic acid-treated liver cancer HepG2 and SNU-449 cells, the LC3II protein was upregulated to induce autophagic death. Most studies believe that autophagy is a double-edged sword [15]. While moderate autophagy protects cells from external stimuli, overregulation of autophagy activity causes autophagic cell death [37]. We found that Sa/DDP inhibited the survival of the liver cancer cells in a dose-dependent manner. However, the Annexin V-FITC/PI staining showed that apoptosis accounts for only a part of the cell death. It was suggested that Sa/DDP-induced death of the liver cancer cells might also depend on another death assay. Some studies have found the antiapoptotic protein Bcl-2 to regulate autophagy by interacting with Beclin-1 [38]. The MDC staining is a specific assay for detecting autophagosome formation, and we observed that the Sa/DDP treatment can activate intracellular autophagy while inhibiting the activity of the HCC cells, thus killing the HCC cells. As marker proteins of autophagy, the Beclin-1, Atg5, and LC3II protein expression increased and p62 protein expression decreased, showing that Sa/DDP treatment can induce the vesicles and lysosomes to combine into autolysosomes and cause macroautophagy, suggesting that Sa/DDP can induce the autophagic death of the liver cancer cells.

Autophagy as a tumor suppressor induces tumor cell death [39]. However, the poor prognosis of various cancer patients is closely related to the high expression of autophagy-related LC3 and Beclin-1 protein [40]. The activation of autophagy under chemotherapeutic conditions has a cytoprotective effect conferring chemotherapy resistance to the tumor cells. Different types of chemotherapy drugs can induce autophagy in the cancerous liver cells, leading to chemoresistance [41, 42]. This is consistent with the mechanism of drug resistance in the tumor cells. The tumor cells excrete the antitumor drugs accumulated in the cell by activating autophagy, acting as tumor-resistant drugs [43]. In the pancreatic cancer PANC-1 cell model treated with gambogic acid, both autophagy and apoptosis can occur. While inhibiting autophagy, the toxic effect on pancreatic cancer PANC-1 cells is enhanced and the apoptosis rate increased [44]. Similarly, treatment of oral cancer Ca922 and SCC2095 cells with ursolic acid can induce the caspase-dependent apoptosis and upregulate the expression of autophagy-related proteins, LC3 and p62. Apoptosis and autophagy may coincide in the antitumor effect caused by ursolic acid, and the apoptotic activity is enhanced after the inhibition of autophagy [45]. We found that, in the Sa/DDP-induced death of the liver cancer cells, autophagy can induce the autophagic death of the liver cancer cells. Concomitantly, the Sa/DDP-induced death can protect the tumors from external stimuli to a certain extent. The inhibition of autophagy activity of the tumor cells and Sa/DDP treatment can enhance the killing effect of DDP on the HCC cells, serving as a potential novel strategy for treating the tumor.

In summary, Sa and DDP in combination can inhibit the proliferation and migration of the HCC cells and induce apoptosis and autophagy, thereby exerting an anti-HCC effect. The possible mechanism of this therapeutic effect is to regulate the expression of the apoptosis-related proteins and activating the caspase-3 protein. However, the intervention of Sa/DDP in the HL-7702 cells showed no apparent cytotoxicity indicating the feasibility of using Sa/DDP as the adjuvant chemotherapy for HCC. In addition, the inhibition of autophagy by HCQ was found to enhance the apoptosis induced by Sa/DDP, suggesting that the combinatorial use with autophagy inhibitors may be a promising therapeutic strategy. Further in vivo and clinical studies are necessary for determining the effectiveness of Sa/DDP in treating HCC.

## 5. Conclusions

The main findings and implications of the work are clearly explained, highlighting its importance and relevance.

## Figures and Tables

**Table 1:** The IC50 values of Sa treatment for 24 h, 48 h, and 72 h in three cell lines.

Cell line	IC50		
	Time		
	24 h	48 h	72 h
HepG2	1795 *μ*M	1379 *μ*M	793.5 *μ*M
SMMC-7721	2580 *μ*M	1625 *μ*M	1351 *μ*M
HL-7702	5056 *μ*M	4431 *μ*M	4020 *μ*M

**Table 2:** The IC50 values of Sa/DDP treatment for 24 h, 48 h, and 72 h in three cell lines.

Cell line	IC50		
	Time		
	24 h	48 h	72 h
HepG2	1515 *μ*M	1002 *μ*M	680.1 *μ*M
SMMC-7721	1951 *μ*M	1642 *μ*M	959.2 *μ*M
HL-7702	4544 *μ*M	3727 *μ*M	3285 *μ*M

## Data Availability

The datasets used and/or analyzed during the current study are available from the corresponding author upon request.
